# Revealing Dynamic Behavior in High Dielectric Poly(thiourethane)-Based Vitrimer-like Materials

**DOI:** 10.1021/acsapm.4c00681

**Published:** 2024-04-27

**Authors:** Federico Guerrero-Ruiz, Itziar Otaegi, Ester Verde-Sesto, Sebastian Bonardd, Jon Maiz

**Affiliations:** † Centro de Física de Materiales (CFM) (CSIC-UPV/EHU)-Materials Physics Center (MPC), Paseo Manuel de Lardizábal 5, 20018 Donostia-San Sebastián, Spain; ‡ POLYMAT and Department of Advanced Polymers and Materials: Physics, Chemistry and Technology, Faculty of Chemistry, University of the Basque Country UPV/EHU, Paseo Manuel de Lardizábal 3, 20018 Donostia-San Sebastián, Spain; § IKERBASQUE-Basque Foundation for Science, Plaza Euskadi 5, 48009 Bilbao, Spain

**Keywords:** covalent adaptable networks, vitrimer-like, poly(thiourethane), glass transition temperature, topology freezing transition temperature, dielectric spectroscopy

## Abstract

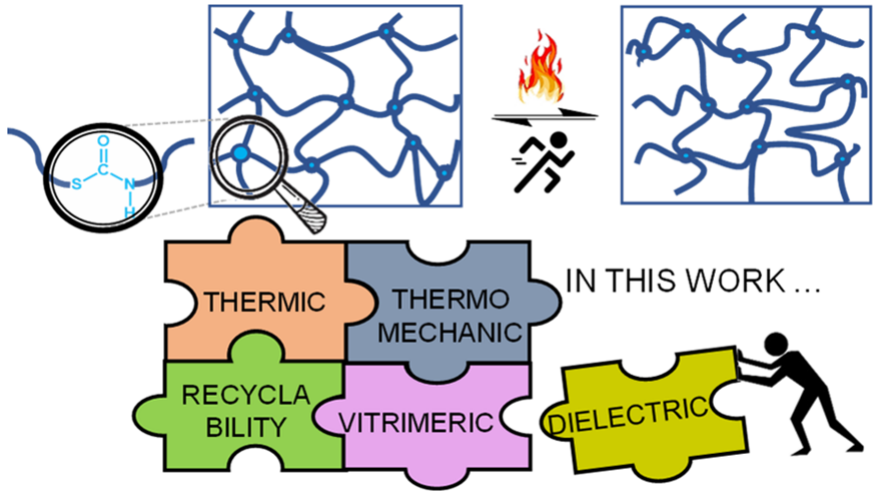

Here, we have explored covalent adaptable networks (CANs) comprising poly(thiourethane)-based systems (PTUs). The PTUs were synthesized through the combination of thiol and isocyanate monomers in stoichiometric proportions, in the presence of dibutyltin dilaurate (DBTDL) as catalyst. Dynamic mechanical analysis (DMA) provided detailed insight into the vitrimeric behavior. Through these investigations, we evaluated the viscoelastic, thermomechanical, and vitrimeric properties. Additionally, broadband dielectric spectroscopy (BDS) revealed the various relaxation processes inherent in such vitrimer-like materials. We methodically examined the evolution of each relaxation in every prepared sample to comprehend the operational mechanisms in these vitrimer-like systems. Our findings underscore that depending on the PTU formulation, the glass transition temperature (*T*_g_) and the topology freezing transition temperature (*T*_v_) can be effectively distinguished and studied. Considering the high dipole moment of the dynamic bonds present in these systems, there is potential for utilizing them as dielectric materials working under the concept of dipolar glass polymers. Furthermore, the reversibility exhibited by their inner chemical structures positions them as promising candidates for active layers in capacitor devices, particularly for energy-related applications, with the ability to be recyclable while maintaining almost invariant both their mechanical and dielectric properties, thus promoting the extension of the lifespan of electronic devices.

## 1 Introduction

The Organization for Economic Co-operation and Development (OECD) forecasts a significant increase in global plastic production, with estimates reaching 516 million tons by 2025, marking a doubling in just 20 years and projected to double again in the next 30 years. Despite efforts, Europe currently recycles only 33% of its plastic, with approximately 10% derived from recycled waste, leaving a significant portion to be incinerated or deposited in landfills. Enhancing the recyclability of plastics is crucial for the sustainability of the industry, making polymer recycling one of the most extensively researched topics today.^1^

The recycling of thermoplastics through mechanical and chemical processes is well-established due to the weak, noncovalent interactions that govern their structure. However, the same cannot be said for thermosets, which are hindered by the presence of strong covalent bonds between polymer chains, preventing their reusability.^2^ Thermosets represent approximately 12% of global polymer production, making their recycling a significant challenge.^3^ Chemical recycling of thermosets involves breaking down the cross-linked network and utilizing the recovered fragments through complex isolation protocols. For example, polyurethanes (PUs) can be degraded via alcoholysis to obtain polyols, while epoxy composites undergo alcoholysis followed by reaction with polyamines.^4,5^ Mechanical recycling of thermosets is even more limited, typically involving grinding the material into particles and incorporating it as an additive in other materials.^6^ A breakthrough in thermoset recycling involves the integration of dynamical covalent bonds into their structure, resulting in covalent adaptable networks (CANs). These networks feature reversible chemical linkages triggered by external stimuli like heat or light, among others, allowing for exchange reactions to occur. Depending on the mechanism of the exchange reaction, CANs exhibit either dissociative or associative behavior. Vitrimers, first reported in 2011, represent materials with associative behavior,^7^ while more recently materials showing a dissociative mechanism, yet with an extremely fast reversion, have been termed “vitrimer-like” systems, presenting a behavior similar to that of an associative mechanism.^8^

These materials offer excellent mechanical and thermal performance akin to thermosets but possess the recyclability of thermoplastics, combining the best attributes of both systems. In vitrimers and vitrimer-like materials, determining the topology freezing transition temperature (*T*_v_) is crucial but a still controversial research subject in the field. *T*_v_ represents the temperature at which the material’s viscosity reaches 10^12^ Pa·s, signifying significant exchange reaction activity.^9^ Classically, as showed in numerous reports, the glass transition temperature (*T*_g_) is lower than *T*_v_, with viscosity being controlled by the kinetics of exchange reactions. Surprisingly, the idea of a material with *T*_g_ > *T*_v_, where viscosity is initially governed by diffusion and subsequently by exchange kinetics, was a hypothesis until it was reported in 2017 by Nishimura et al.^10^

Poly(thiourethane)s (PTUs), analogs of PUs with a sulfur atom, exhibit superior exchange kinetics and show promise in optical and biomedical applications.^11^ Despite their advantages, research on PTU-based CANs is limited compared to PUs. PTUs are synthesized through the condensation of isocyanates and thiols, resulting in materials with dynamic properties facilitated by thiourethane–thiourethane or thiol–thiourethane exchange reactions.^12^ Since the initial report in 2019 by Torkelson’s group, various thiol–isocyanate systems with dynamic properties have been investigated, employing different catalysts and exploring sustainable alternatives.^13−16^ In the quest for more sustainable materials, there have been reports on substituting monomers derived from biomass for those derived from fossils, along with the development of non-isocyanate poly(thiourethane)s (NIPTUs).^17,18^ Additionally, dynamic PTUs have been employed as a polymeric matrix in nanocomposites.^19^

From an experimental perspective, tracking bond exchange reactions and observing the topological transition pose significant challenges. In recent years, several research groups have explored various experimental techniques to address these challenges. Dynamic thermomechanical (DMA) studies are commonly used to determine the *T*_v_. Values for *T*_v_ are typically extrapolated from data obtained through stress relaxation and creep tests. However, this method may lack precision as it involves indirect measurement. Alternatively, *T*_v_ can be also estimated using dilatometry and static thermomechanical (TMA) studies of the coefficient of thermal expansion.^20^ However, the application of external force in these techniques can introduce tension on the covalent bonds, potentially shifting the transition.

Researchers have also explored static methods for *T*_v_ determination. Yang et al. proposed the use of aggregation-induced-emission (AIE) luminogens, which measure the difference in fluorescence below and above *T*_v_ without applying external forces.^21^ Similarly, Arbe et al. employed scattering techniques to determine the topological transition temperature in polyisoprene-based vitrimers. By using suitable thermal protocols, they accurately revealed this transition.^22^ Additionally, broadband dielectric spectroscopy (BDS) is a valuable technique for studying polymer dynamics and understanding the bond exchange mechanism.^23^ In this study, we will utilize a variety of characterization techniques to demonstrate their combined accuracy in determining *T*_v_ and understanding bond exchange reactions.

Beyond their utility in determining topological transitions, CANs offer the potential for self-healing, enabling structural restoration and functional recovery.^24^ This innovation has significant implications for enhancing the reliability and extending the lifespan of materials, particularly in electronic applications.^25^ For example, the demand for polymers with high dielectric constants and low dissipative behavior is increasing in emerging applications such as gate dielectrics for printable electronics, soft robotics, power-pulse circuits, and polymer film capacitors. Achieving higher dielectric constants in polymers involves leveraging orientational polarization from molecular dipoles, thereby enhancing their performance in energy-related applications.

To address these challenges, we propose creating dipolar glass polymers (DGPs), specifically referred to as dipolar glass vitrimers (DGVs) in this case. DGPs exhibit similarities to spin glasses found in magnetic materials.^26^ Within a DGP, mobile dipole groups are confined within the free volume of a glassy polymer matrix. Utilizing sub-*T*_g_ transitions as orientational motions for polarization, DGPs enhance the dielectric constant, potentially resulting in narrow electric displacement–electric field (D-E) loops with relatively high dielectric constants. Moreover, due to frozen chain dynamics below *T*_g_, DGPs may demonstrate low dielectric loss.

In this study, we introduce an alternative and widely recognized experimental approach for determining and estimating the temperature range where the *T*_v_ transition occurs in vitrimer-like materials. BDS emerges as a valuable tool in characterizing these systems, effectively discerning the various relaxation processes within the material. Through this method, we can accurately distinguish between *T*_g_ and *T*_v_ processes when they are properly separated in the temperature scale. Our investigation employed a series of model vitrimer-like systems based on PTUs, with *T*_v_ determined through DMA and visualized BDS, while *T*_g_ was assessed using differential scanning calorimetry (DSC), DMA, and BDS. Furthermore, we explore the potential of these materials as DGPs, given their high dipole moment entities, which exhibit competitive dielectric constants and low loss factors, thereby positioning them as relatively high dielectric polymer systems with low dissipative behavior. The reversible and recyclable nature of these materials makes them promising candidates for various energy-related applications.

## 2 Experimental Procedures

### 2.1 Reagents

Trimethylolpropane tris(3-mercaptopropionate) (S3, ≥95.0%), pentaerythritol tetrakis(3-mercaptopropionate) (S4, >95.0%), hexamethylene diisocyanate (HDI, >98.0%), and dibutyltin dilaurate (DBTDL, synthesis grade) were obtained from Aldrich. 2,4-Toluene diisocyanate (TDI, 80% with the remaining 20% being 2,6-toluene diisocyanate) was sourced from Thermo Scientific. All products were used as received.

### 2.2 Formulation and Material Preparations

**Figure 1:**
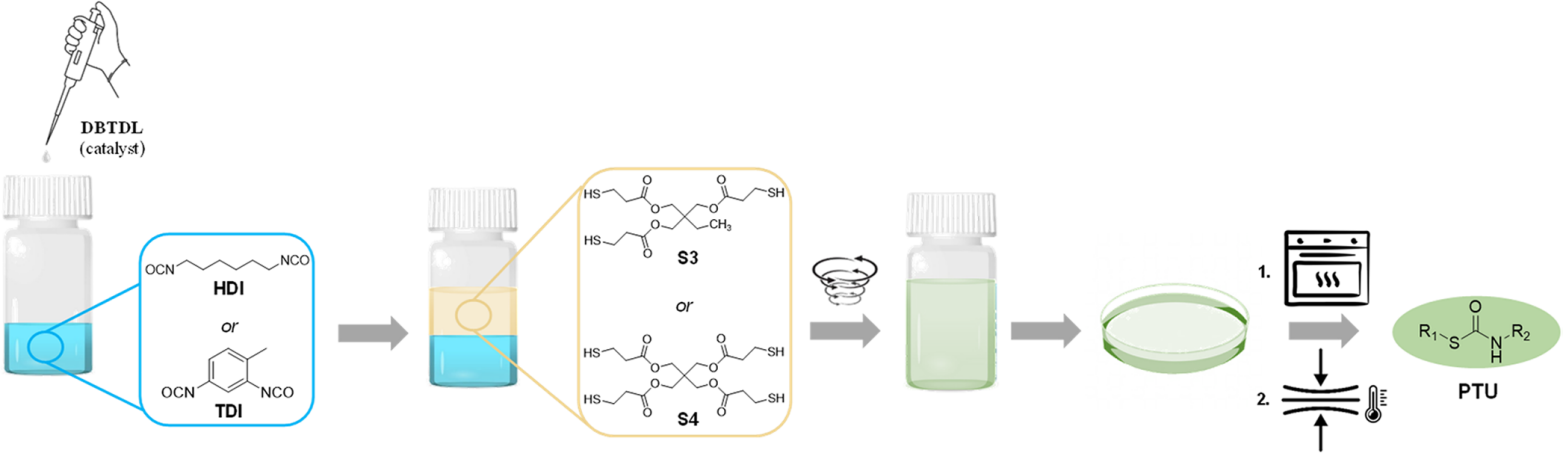
Schematic illustration of the preparation method for vitrimer-like poly(thiourethane) systems, PTUs.

The thiol and isocyanate monomers were mixed in stoichiometric proportions (1 mol of thiol group per mol of isocyanate group) and contained a quantity of DBTDL catalyst equivalent to 1 mol % of the total of theoretical thiourethane groups. The composition and nomenclature of the prepared formulations are shown in Table S1. Additionally, Figure 1 depicts the structures of monomers and the finally produced material. Furthermore, the film preparation procedure is schematically illustrated in the same figure. First, the catalyst is added to the isocyanate, followed by the addition of thiol. The formulation is then rapidly mixed under vigorous manual stirring, and the resulting mixture is placed in a Teflon Petri dish. The mixture is precured in an oven at 373 K for 1 h and then hot-pressed at 453 K for 2 h under a pressure of 20 bar, between two aluminum plates covered with Teflon. Transparent, thin, and homogeneous films were obtained in all cases. Finally, the film is die-cut to obtain dog-bone-shaped samples following the ASTM D638 (type V) standards. The samples have an average thickness of 0.25 mm.

### 2.3 Characterization Techniques

Thermal gravimetric analysis (TGA) measurements were conducted using a Q500-TA Instruments apparatus, with a heating rate of 10 K/min under a nitrogen flow of 50 mL/min, from room temperature to 1073 K. For each measurement, approximately 10 mg of samples were placed in platinum pans. The analysis of the recorded thermograms allows the obtainment of the degradation onset temperature (*T*_i_), maximum weight loss temperatures (*T*_peak_), and residue percentage values. For the sake of clarity, *T*_i_ values were defined as the temperature at which the material loses 5% of its initial weight, while *T*_peak_ values were extracted from the maxima of DTGA curves.

Differential scanning calorimetry (DSC) measurements were performed using a Q2000 TMDSC instrument. Nitrogen gas with a flow rate of 50 mL/min was employed. Samples (approximately 10 mg) were encapsulated in aluminum pans and subjected to a specific thermal treatment: cooled to 223 K from 473 K, held at this temperature for 5 min, then heated at a rate of 20 K/min to 473 K, held at this temperature for 5 min, and then cooled at 20 K/min to 223 K.

The viscoelastic, thermomechanical, and vitrimeric properties were assessed using a DMA Q800 analyzer from TA Instruments equipped with a film tension clamp. Properties were extracted from tan δ, stress relaxation, and creep-recovery tests.

The evolution of tan δ and dynamic modulus with temperature was analyzed. The samples were subjected to testing at a heating rate of 5 K/min, ranging from 273 to 363 K, with a frequency of 1 Hz and a strain of 0.1%.

In the stress relaxation test, the sample was heated to 433 K for 5 min, followed by the application of a constant strain of 1.5% over the sample, with the stress level measured for 30 min. The stress–relaxation cycle was then repeated every 5 K until reaching 473 K. The relaxation stress (σ) was normalized to the initial stress (σ_0_), and the characteristic relaxation time (τ*) was determined as the time necessary for 37% (100/e) of the initial stress to relax. The obtained relaxation times at each temperature were used to calculate activation energy values (*E*_a_) using an Arrhenius-type equation:
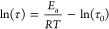
1

In the creep test, the sample was heated to the desired temperature for 5 min and then subjected to stretching under a stress of 0.1 MPa for 30 min. To determine the viscosity of the material, a series of creep tests were conducted, ranging from 433 to 473 K with an increment of 5 K for each test. The viscosity (η) at each temperature was obtained from the linear part of the creep curves (dε/d*t*) using the following equation:
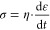
2

The *T*_v_ was determined through stress relaxation and creep tests (temperature for a viscosity of 10^12^ Pa·s). To determine *T*_v_ in the stress relaxation test, both eq 1 and the Maxwell equation (eq 3), which correlates viscosity (η) with the characteristic relaxation time (τ*), were employed, where *E*′ represents the storage modulus in the rubbery state (assuming *E*′ to be relatively invariant in this state):
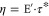
3For the creep test to determine the *T*_v_ value, eq 2 was employed.

Broadband dielectric spectroscopy (BDS) experiments were conducted using a Novocontrol Alpha high-resolution analyzer, covering a frequency range from 10^–1^ to 10^7^ Hz with an applied AC voltage of 1.0 V. Bubble-free film samples were placed between two circular gold-plated electrodes of different diameters (20 mm) in a parallel plate capacitor configuration, with the larger diameter electrode serving as the bottom part. Isothermal experiments were performed at intervals of 10 K, ranging from 475 to 125 K, with temperature controlled by a cryostat (BDS 1100) exposed to a heated gas stream evaporated from a liquid nitrogen container.

## 3 Results and Discussion

### 3.1 Thermal Stability of Materials

The thermal decomposition profiles of the previously prepared samples, along with their first derivative (DTGA), are shown in Figure S1 in the Supporting Information. Additionally, the main data collected from the TGA analysis for all samples are summarized in Table S2.

All materials exhibit *T*_i_ values above 523 K,^27^ which is associated with the robust cross-linked structure of samples along with the presence of strong hydrogen bonds established between thiourethane groups (with a dissociation energy measured in simple molecules of 21.7 kJ/mol). However, the thermal stability of poly(thiourethane)s is limited to not exceed 573 K due to the thermal reversion of the thiol–isocyanate bond. The nature of nitrogen and thiol substituents strongly influences the degradation temperature of materials. In our case, the materials prepared from aliphatic diisocyanates exhibit higher thermal stability compared to those prepared from aromatic diisocyanates. Generally, aromatic polymers demonstrate better thermal resistance than aliphatic ones. However, in these poly(thiourethane)s, the degradation process would begin with the thermal reversion of the thiourethane group, so the *T*_i_ would depend on its equilibrium with the starting groups. Notably, we observed a similar decomposition trend to that reported in polyurethanes: alkyl-NCO/alkyl-OH > aryl-NCO/alkyl-OH > alkyl-NCO/aryl-OH > aryl-NCO/aryl-OH.^28^ Moreover, a greater thermal resistance can be observed in materials prepared from tetrathiol (S4) compared to those prepared from trithiol (S3), probably ascribed to a higher degree of cross-linking resulting from a higher thiol functionality.

The presence or absence of a catalyst also has a significant influence on the thermal stability of materials. Catalysts used in the curing process and/or exchange reactions tend to influence importantly the degradation process, usually leading to lower *T*_i_ values. Gamardella et al. reported that poly(thiourethane)s prepared using 1,8-diazabicyclo[5.4.0]undec-7-ene (DBU) as a base catalyst degrade earlier than those prepared using an acid catalyst (DBTDL), even when the latter has a concentration 100 times higher.^15^ Due to the above, DBTDL was the catalyst of choice in this work.

The DTGA curve shows that thermal degradation of materials takes place in three consecutive and well-differentiated steps. The first step, centered around 573 K, would be triggered by the thermal-activated reversion of thiourethanes, promoting the start of the volatilization of species. In this regard, it is feasible that this step is dominated, to some extent, by the elimination of fractions of HDI and TDI moieties, structures that register boiling points within this temperature interval. In materials prepared from aromatic diisocyanates, this first peak appears at lower temperatures than in those prepared from aliphatic diisocyanates, due to the previously mentioned decomposition order. The second stage, at about 623 K, is associated with the decomposition of the ester bond in the structure of thiols.^15^ When a base catalyst is employed, this peak appears well-differentiated from the first one. However, in materials prepared with an acid catalyst (as in this case), both peaks appear overlapped. The breakdown of the ester bond seems to be facilitated in HDI materials compared to TDI materials, with a significant difference of about 25 K, because the aromatic structure hinders the β-elimination reaction.^29^ The last step, appearing in the range 705–725 K, could be associated with the final fragmentation and volatilization of the remaining material produced after the two previous decomposition stages.

### 3.2 Glass Transition Temperature (*T*_g_) Determination by DSC and DMA Techniques

**Figure 2:**
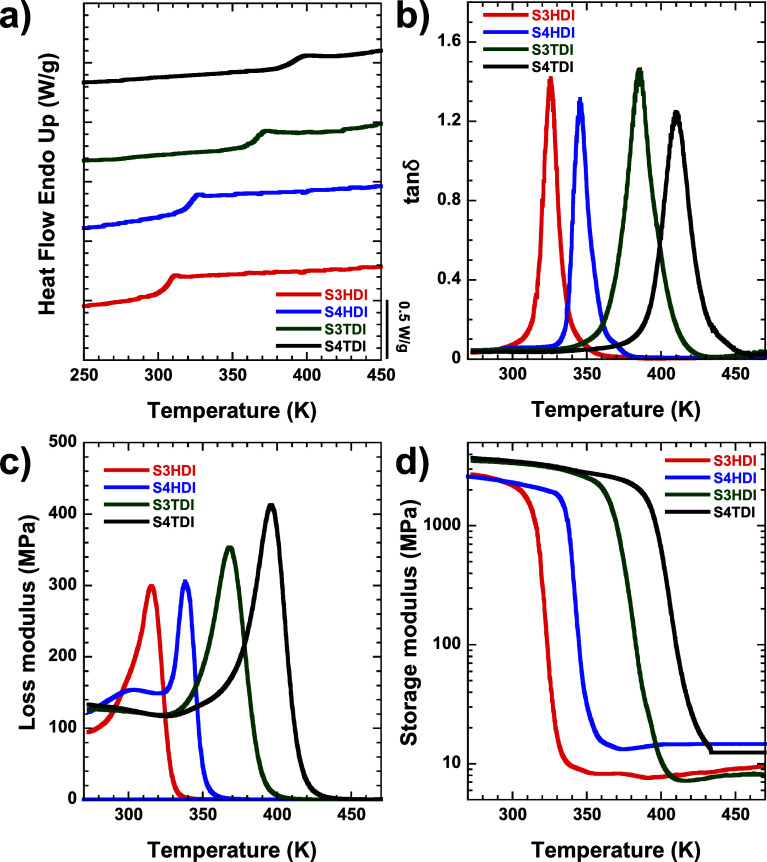
(a) DSC heating scan at 20 K/min after the previous cooling process, (b) loss tangent, (c) loss modulus, and (d) storage modulus at 1 Hz for the S3HDI (red), S4HDI (blue), S3TDI (green), and S4TDI (black) samples.

Different techniques can be employed to determine this transition in vitrimer-like materials, with DSC and DMA being the two most commonly used. Through DSC, we define *T*_g_ values as the onset temperature of the observed transition. Conversely, in DMA analysis, the *T*_g_ value is extracted separately from tan δ, loss modulus (*E*″), and storage modulus (*E*′) profiles following already established protocols.^30^ In tan δ and *E*″ profiles, *T*_g_ values are easily defined as the peak’s maximum, while in *E*′ profiles, the *T*_g_ value is obtained from the intersection point between the linear fits arising from data belonging to the glassy plateau and the sudden drop during the viscoelastic regime.^31^ The results obtained for the studied systems are shown in Figure 2 and summarized in Table 1.

**Table 1:** *T*_g_ Values Extracted from DSC and DMA Analysis and Main Data Obtained from tan δ of Prepared Materials

	DSC	DMA					
material	*T*_g_^a^ (K)	tan δ^b^ (K)	*E*′^c^ (K)	*E*″^d^ (K)	*E*_glassy_^′^^e^ (MPa)	*E*_rubbery_^′^^f^ (MPa)	fwhm^g^ (K)
S3HDI	308	326	315	317	2702	9	12
S4HDI	323	345	337	339	2875	12	12
S3TDI	367	386	368	369	3504	8	22
S4TDI	393	410	395	397	3724	12	22

a Onset temperature of the step of the heat flow as a function of the temperature curve.

b Maximum peak of tan δ as a function of the temperature curve.

c Maximum peak of the loss modulus as a function of the temperature curve.

d Onset temperature of the line from the glassy-plateau and a line from the sudden drop determined by TA Instrument software in storage modulus as a function of the temperature curve.

e Glassy storage modulus determined as *T*_tanδ_ – 50 K.

f Rubbery storage modulus determined as *T*_tanδ_ + 50 K.

g Full width at half-maximum of the tan δ peak.

From Table 1, it can be observed that all *T*_g_ values fall within the same order of magnitude, with subtle differences depending on the employed technique (DSC or DMA) and the applied definition in DMA analysis (tan δ, *E*′, or *E*″). In this context, the lowest recorded values were obtained using DSC, while the highest were measured from the maxima of the tan δ curves (DMA). Nonetheless, the overall difference between both methods corresponds to approximately 20 K, which can be explained by temperature calibration errors, the influence of the heating rate, and the external force applied.^32^

The *T*_g_ values obtained by both techniques follow a common order: S3HDI < S4HDI < S3TDI < S4TDI. The higher *T*_g_ observed in samples made from S4 (compared to those made from S3) can be explained in terms of a higher number of cross-linking points, which would favor the formation of a more reticulated structure. Meanwhile, the higher *T*_g_ of TDI materials compared with HDI materials can be derived from their structural nature, where TDI constitutes a hard (rigid) fragment, whereas HDI constitutes a soft (flexible) fragment. Importantly, the specimen S4TDI exhibited the highest *T*_g_ value.

Additional information about the thermomechanical behavior of the materials can be obtained from tan δ experiments, as collected in Table 1.

The glassy storage modulus is strongly influenced by the functionality of the thiol and the structure of the isocyanate. Higher glassy storage modulus values were reported in S4 materials compared to S3 materials and in rigid fragment materials compared to soft fragment materials, as previously reported by Guerrero et al.^14^ The rubbery storage modulus is more influenced by the cross-linking degree of the network, being higher in S4 materials than in S3 materials due to the higher functionality of the thiol.

The full width at half-maximum (fwhm) values indicate a high degree of homogeneity in the materials, attributed to the click nature of the thiol–isocyanate reaction when DBTDL is employed as a catalyst.^13^ Despite this, materials made from TDI present wider tan δ peaks than those made from HDI, possibly owing to the use of a mixture of TDI isomers (2,4- and 2,6-toluene diisocyanate) as the starting product.

### 3.3 Topology Freezing Transition Temperature (*T*_v_) Determination

The topology freezing transition temperature can be determined using various techniques such as TMA, AIE, and scattering techniques. However, the most commonly employed technique is DMA in different modes, mostly including stress relaxation and creep tests. In this work, *T*_v_ values have been determined by these two methods, and the results are presented in Table S3.

**Figure 3:**
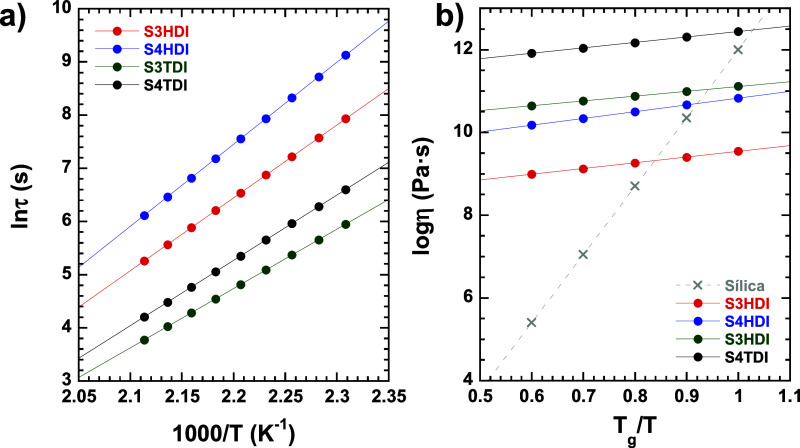
(a) Arrhenius plot of characteristic relaxation time against inverse temperature and (b) Angell fragility plot for the S3HDI (red), S4HDI (blue), S3TDI (green), and S4TDI (black) samples including vitreous silica (gray) as a reference of an ideal strong glass former material.

The difference between the temperatures obtained by both methods ranges from 3 K in S3HDI to 21 K in S3TDI. In both cases, the temperature has been determined by extrapolating relaxation time and viscosity trends, where both variables become unmeasurable within reasonable times. The assumption of linear behavior in the extrapolated region, coupled with experimental imprecisions such as instrument calibration errors due to temperature variations and changes in specimen dimensions caused by thermal expansion and applied forces during the assay, among others, can result in errors exceeding 100 K.^33^ Nevertheless, we can consider the temperatures obtained from both tests to be quite similar and, most importantly, following similar trends. In this context, both experiments revealed differences between the obtained materials. This could be ascribed to a higher local concentration of thiourethane groups varying the kinetics of the exchange process, as *T*_v_ has shown to be governed by diverse factors within them the density of exchangeable cross-links and abundancy of chemical functionalities.^34^ Aiming to complement this part of the characterization, additional information about the thermomechanical behavior is represented in Figure 3.

From Figure 3a, and using eq 1, the activation energy (*E*_a_) associated with the exchange reaction can be calculated, resulting, in this case, in the obtainment of values falling within the same range but following clear tendencies. In this regard, it draws attention to the difference existing between specimens containing TDI and HDI moieties, which has been previously addressed by Gamardella et al., arguing that the aromaticity of TDI has a positive effect on the kinetics of the exchange reaction, leading to shorter relaxation times as also shown in Table 2.^35^

**Table 2:** Main Data Obtained from Stress Relaxation and Creep Tests on Prepared Materials

	stress relaxation			creep	
Material	*E*_a_^a^ (kJ/mol)	*r*^2^	τ*^b^ (min)	*E*_a_^a^ (kJ/mol)	*r*^2^
S3HDI	114	0.9999	3.2	104	0.9982
S4HDI	129	0.9999	7.5	123	0.9994
S3TDI	93	0.9982	0.7	83	0.9997
S4TDI	102	0.9982	1.1	99	0.9995

a Activation energy of the exchange reaction.

b Time to reach a σ/σ_0_ = 0.37 at 473 K.

In addition, from Figure 3b, the Angell fragility plot provides a measure of how the dynamic nature of a material changes as the temperature approaches the *T*_g_. This plot aids in classifying materials into two categories: “strong glass”, where the relationship between viscosity and temperature follows an Arrhenius model, and “fragile glass”, where the Arrhenius model does not apply. Thermoplastics and dissociative CANs belong to the latter group, while associative CANs (vitrimers) and vitrimer-like materials exhibit a “strong glass” behavior, akin to silica, represented in Figure 3b as a reference for an ideal strong liquid.^36^ In our case, all the systems can be classified as “strong glass” materials.

**Figure 4:**
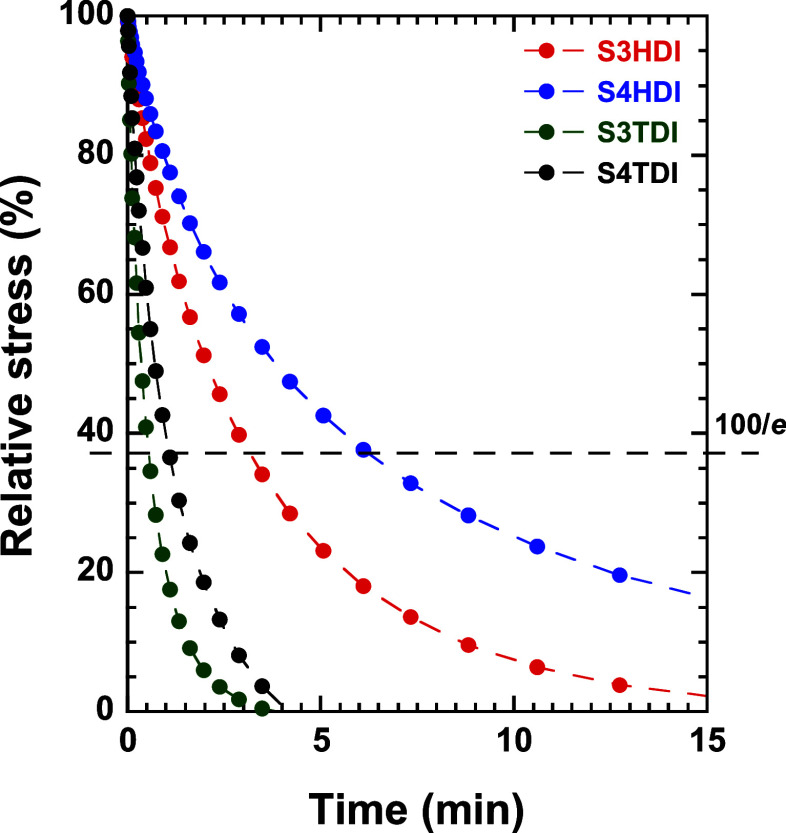
Relative stress relaxation curves as a function of time at 473 K for the S3HDI (red), S4HDI (blue), S3TDI (green), and S4TDI (black) samples. The dashed line represents the relative stress value used as a reference for the calculation of the characteristic time.

Over the past few years, Serra and co-workers^14−17,19,35^ have shown that these materials exhibit vitrimeric-like behavior, characterized by the dissociation of the thiourethane group into isocyanate and thiol. This process is followed by rapid reformation, resulting in a decrease in viscosity upon heating, following an Arrhenius-type evolution. Figure 4 shows the relative stress as a function of time at 473 K, where it is possible to observe a highlighted difference between materials prepared from aliphatic and aromatic diisocyanates, with the latter exhibiting total relaxation in less than 5 min. The comparison of the relative stress of materials prepared from thiols of different functionalities shows shorter relaxation times in materials prepared from S3 due to a lower cross-linking degree and higher mobility of the polymeric chains, making the exchange reaction more favored. All materials exhibit low characteristic relaxation times at 473 K, with those prepared using TDI moieties achieving complete relaxation before the end of the experimental time.

### 3.4 Analysis of Dielectric Properties. Determination of Topological Freezing Temperature (*T*_v_)

**Figure 5:**
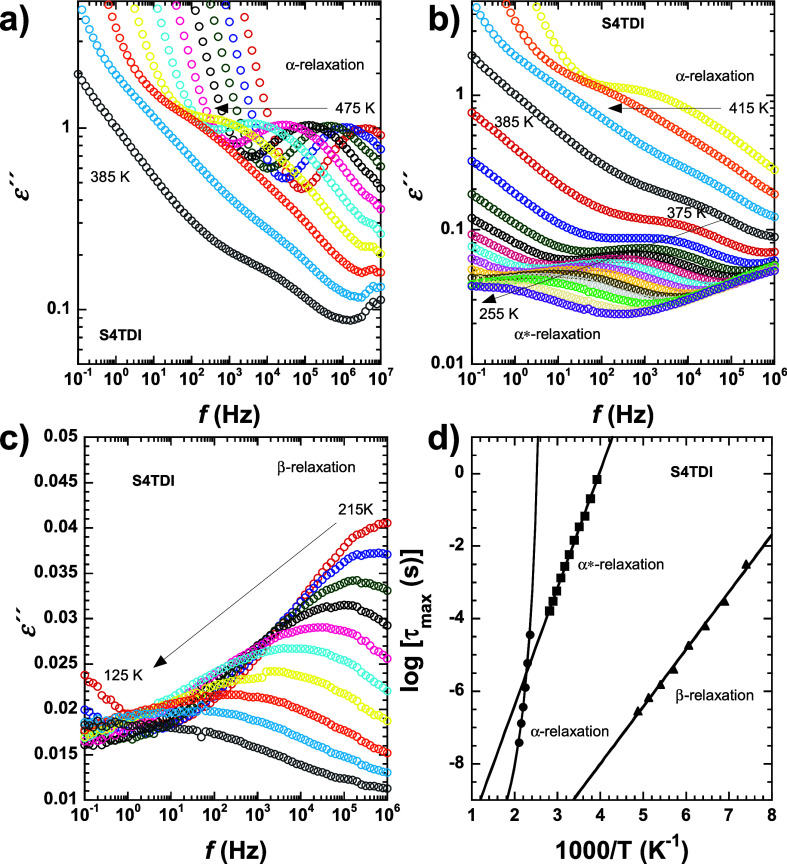
Isothermal plots of ε″ for the S4TDI sample (a) at high temperatures, (b) at intermediate temperatures, and (c) at low temperatures. The characteristic times obtained from the maxima of the loss peaks are represented in panel d for the different relaxation processes observed (α-relaxation: filled circles; α*-relaxation: filled squares; β-relaxation: filled up-triangles).

As previously stated, these vitrimer-like systems are highly attractive as functional materials due to their robust mechanical properties, similar to thermosets, but with the advantage of being recyclable and self-healing, akin to thermoplastics. In this context, owing to the presence of high-dipole moment thiourethane moieties, we devised these materials as dielectrically active layers for application in energy storage, motivating us to pursue their dielectric characterization. Consequently, through BDS, we seek to first evaluate the dynamics of these reversible networks, which is essential for comprehending the main properties of these materials. As a first approach, isothermal experiments of the dielectric loss parameter (ε″) were carried out in the frequency range from 10^–1^ to 10^7^ Hz and recorded in the temperature range of 475–125 K. The ε″ isotherm spectra of the vitrimer-like material S4TDI, reported here for the first time, are exhibited in Figure 5, while the data belonging to the other systems can be consulted in the Supporting Information file (Figures S2, S3, and S4).

Figure 5 presents the data collected for the different relaxation processes observed within the studied temperature range. Figure 5a represents the high-temperature regime (475–385 K), where, at high frequencies, a first relaxation process can be observed, denoted as α-relaxation. According to our previous DSC and DMA experiments, the *T*_g_ value of this material falls within this range of temperatures, and considering the features exhibited by this transition in terms of frequency and temperature dependence, we ascribe this α-relaxation to the long-range segmental motions of polymer chains typically associated with the *T*_g_ phenomenon. Importantly, it is worth noting that at low frequencies, tracking this process becomes difficult due to the appearance of conductivity phenomena. Later, as the temperature decreases, another process begins to emerge at high frequencies, labeled here as α*-relaxation. This relaxation, as shown in Figure 5b, becomes detectable particularly when the temperature changes from 395 to 375 K. At this point, a distinct and broader process emerges, remaining visible until 255 K. In a similar manner to before, DMA experiments reveal that the *T*_v_ of this specimen appears in this range of temperatures, and therefore, we find it feasible to relate this α*-relaxation to the unlocking of new dynamical motions within the systems triggered by the activation of thiourethane bond exchange reactions. Upon further temperature decrease, a secondary relaxation denoted here as β-relaxation is observed, as visualized in Figure 5c. This relaxation is even broader than the previous one, and these processes typically involve short-range motions of lateral or short chain segments. In this case, we attribute this relaxation probably to reorientational motion performed by dipolar structures present in both dithiourethane and S3/S4 species. It is worth mentioning that for the sample S3HDI, just two relaxation processes are observed, as evidenced in the Supporting Information Figure S2. This could be attributed to the lower *T*_g_ value shown by this specimen, being notably below its measured *T*_v_. Thus, in this sample, conductivity phenomena take place much earlier on the temperature scale when compared with its counterparts, affecting the proper analysis of relaxation phenomena. Nevertheless, in all other cases, three main relaxation processes are observed, as verified in Figures S3 and S4. From all these peaks, the relaxation map can be constructed by extracting the relaxation time from the maximum of each peak in each isothermal plot. This can be calculated as
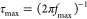
4The obtained data has been represented in Figure 5d for the S4TDI sample, while for specimens S3HDI, S4HDI, and S3TDI, their relaxation maps can be consulted in Supporting Information Figures S2d, S3d, and S4d, respectively.

The differences observed between the *T*_g_ and *T*_v_ values, which have been previously investigated through various experimental techniques, allow us to effectively discern the relaxation processes in the S4TDI sample using BDS. This material exhibits a *T*_g_ value of 393 K as determined by DSC and 410 K as determined by DMA (tan δ). Additionally, the *T*_v_ value calculated by DMA was 371 K based on stress relaxation experiments. In contrast, for the remaining samples, the differences observed between the *T*_g_ and *T*_v_ values are not as distinct as in the S4TDI sample. In some cases, when the *T*_g_ and *T*_v_ fall within the same temperature range, the bond exchange process is observable, and distinct maxima can be identified, as demonstrated in Figures S3 and S4. However, if the *T*_v_ exceeds the *T*_g_ value, this process is usually masked by the conductivity observed at temperatures higher than *T*_g_, as illustrated in Figure S2.

As previously stated, the constructed relaxation map reveals three distinct processes in most of the cases studied herein. The α-relaxation is strongly connected to the *T*_g_ of polymers, and the molecular motions involved in this process depend drastically on temperature. It does not follow the typical Arrhenius temperature dependence; instead, the experimentally observed non-Arrhenius behavior can be approximated by the empirical Vogel–Fulcher–Tammann (VFT) formalism:^37−40^
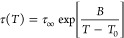
5

**Figure 6:**
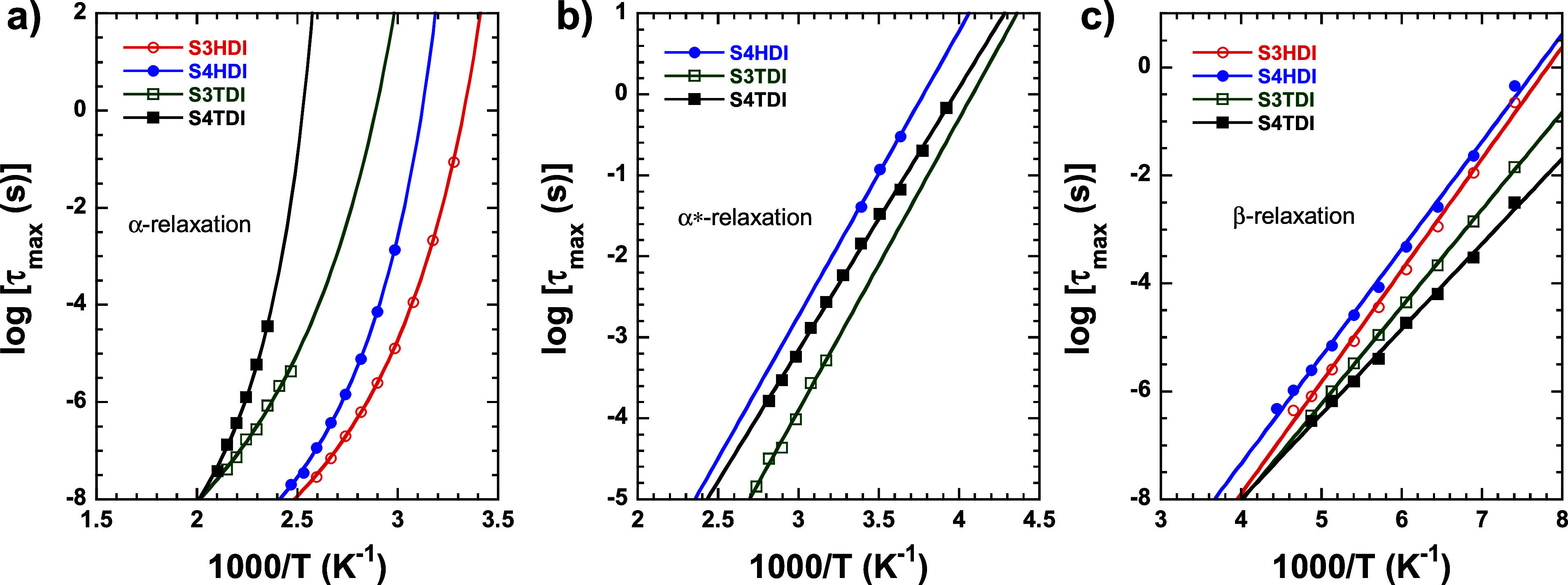
Relaxation map for the (a) high temperature range (α-relaxation process), (b) intermediate temperature range (α*-relaxation process), and (c) low temperature range (β-relaxation process) for the S3HDI (red unfilled symbols), S4HDI (blue filled symbols), S3TDI (green unfilled symbols), and S4TDI (black filled symbols) samples.

In this context, *T*_0_ represents the Vogel temperature, which is the temperature at which the characteristic relaxation time would diverge. *B* is an energetic term connected with the fragility parameter, expressed as *B* = *D* × *T*_0_. This *D* fragility parameter from the VFT law can be correlated with the dynamic fragility parameter, denoted as “*m*” in Angell’s criteria,^36^ enabling the classification of the system as fragile or strong. When *D* from the VFT law has a high value, *m* is low, implying a low fragility material, and vice versa. Finally, τ_∞_ corresponds to the typical vibration frequency, usually fixed between 10^–12^ and 10^–14^ s in polymers. These two parameters can be connected by the following equation:
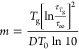
6On the other hand, the analysis of the temperature dependence of the relaxation times of the other two relaxation processes is well-fitted using the Arrhenius equation:
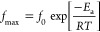
7where *f*_max_ refers to the maximum frequency, *f*_0_ is a pre-exponential term, *E*_a_ is the activation energy, and *R* is the ideal gas constant. It is important to note that the relationship between frequency and time is given by eq 4. With this in mind, the obtained relaxation maps for each specimen and their respective transitions are shown in Figure 6, while Table 3 summarizes the different parameters extracted from the fitting process.

**Table 3:** Fitting Parameters of the α-Relaxation with the VFT Function and α*-Relaxation and β-Relaxation with the Arrhenius Equation for the Different Materials Studied

	α-relaxation				α*-relaxation	β-relaxation
material	*T*_g_^a^ (K)	*B* (K)	*T*_0_ (K)	*m*^b^	*E*_a_ (kJ/mol)	*E*_a_ (kJ/mol)
S3HDI	295	2080 ± 88	271 ± 7	68		40 ± 1
S4HDI	314	1296 ± 12	273 ± 1	117	67 ± 4	38 ± 1
S3TDI	337	2080 ± 86	271 ± 7	80	69 ± 4	34 ± 1
S4TDI	389	1406 ± 38	345 ± 3	132	62 ± 1	30 ± 1

a By extrapolating τ_max_ to τ = 100 s, the *T*_g_ from the dielectric measurements are estimated.

b The values used for the dynamic fragility calculation are τ_*T*_g__ ≈ 10^2^ s and τ_∞_ ≈ 10^–12^ s.

Figure 6a compares the processes related to the segmental dynamics of each sample with respect to the α-relaxation. As previously determined, materials prepared from TDI moieties exhibited higher *T*_g_ values compared to their counterparts bearing HDI. The same trend was observed when comparing specimens constructed with S3 and S4 thiols, with the latter also showing higher *T*_g_ values. Both results find explanation in the “hard segment” nature provided by TDI and in the higher number of cross-linking points generated when using S4, favoring the formation of a more reticulated structure. Thus, Figure 6a illustrates, graphically, how τ_max_ for each specimen depends on temperature. In this sense, it can be verified that to reach a certain τ_max_ value, specimens prepared from TDI will require higher temperatures than those containing HDI, a trend that effectively matches their *T*_g_ values. On the other hand, a more in-depth analysis allows us to state that the evolution of τ_max_ with temperature seems to be closely related to the thiol structure, where specimens containing S3 moieties show a more subtle variation of τ_max_ with temperature than S4 samples. This is represented by the lower *m* values (dynamic fragility) calculated for S3HDI and S3TDI (Table 3), possibly relating this fact to the degree of reticulation that the networks present, becoming relevant when the systems transition from the S3 to S4 structure. Interestingly, and as reported by previous works,^23^ this reticulation would also affect the bond exchange process and, with that, the dynamics of the α*-relaxation (depicted in Figure 6b). As previously demonstrated, the calculated *T*_v_ values are not significantly different from each other, and in three of the four samples studied, the *T*_v_ < *T*_g_ condition is fulfilled, explaining their observability by BDS. Indeed, the sample S4TDI, with the highest *T*_g_ value, exhibited the best-resolved α*-relaxation in BDS compared to the other samples. In addition, it draws attention to the remarkably similar activation energies calculated for this relaxation among the samples. The above could be explained by the fact that the vitrimeric behavior of all samples relies on the same type of dynamic bonds (thiourethanes), and together with their notably similar network structures, the energetic requirements to trigger the exchange reactions should be around the same. On the other hand, the secondary relaxation, denoted as β-relaxation and depicted in Figure 6c, shows more deviations depending on the nature of the sample. As the *T*_g_ value increases, the activation energy of this secondary relaxation decreases.

Another way to showcase dielectric data is through isochronal representations. The advantage of this type of measurement relies on the easiest visual assessment of relaxation phenomena taking place in the material as temperature varies. Figure S5 presents isochronal spectra of the prepared materials recorded at three different frequencies, allowing the identification of the β-, α*-, and α-relaxations on the temperature scale. Fortunately, in these experiments, the presence of the α*-relaxation, related to the vitrimeric behavior, can be successfully confirmed within the range of temperatures where *T*_v_ was determined in Figure S5d. However, for the remaining samples, a less distinct process is observed in this temperature range, as depicted in Figure S5. As explained previously, this disparity may be attributed to the temperature difference observed between the values of *T*_v_ and *T*_g_ and the early contribution of conductivity phenomena.

After these studies, we have corroborated that the obtained materials, especially S4TDI, meet the basic requirements to be considered suitable dielectric materials, fitting within the classification of dipolar glass polymers. Specifically, S4TDI, presenting the highest *T*_g_, exhibited the broadest temperature interval among the specimens where a highly polarized state, mainly ascribed to β-relaxations, and low dissipative behavior were achieved. In this regard, the presence of high dipole moment thiourethane units allowed these materials to reach dielectric constant (ε_r_′) values ranging from 3.8 to 4.8 at 1 kHz at room temperature, with loss factors lower than 0.01, thus qualifying as low-dissipative materials. As expected, the highest ε_r_′ belongs to S4TDI and would be due to the higher local concentration of dipolar structures. In addition to the adequate dielectric properties, we realize that this system can be a competitive material due to its vitrimeric properties that endow it with the ability to be recycled and reused without noticeable loss of properties, as will be shown in the next section.

### 3.5 Recycling Test

To confirm the recyclability of the materials prepared, we selected the S4TDI due to not having been previously reported and presenting the higher ε_r_′ and lower tan δ. The material was ground and hot-pressed at 20 MPa and 473 K for 2 h, conditions that combine a fast and complete relaxation process observed by DMA and the thermal stability observed by TGA. The recycled material was tested by DMA to assess its thermomechanical properties. Figure S6 shows the evolution of tan δ and storage moduli with temperature for the original and recycled S4TDI, where no significant differences can be observed. This confirms that no structural changes and thermal degradation processes to the polymeric networks occurred during the recycling process. Additionally, the *E*_glassy_^′^ value obtained for the recycled S4TDI sample was 3489 MPa, and the *E*_rubbery_^′^ value was 11 MPa. With these values obtained, we can confirm a recycling percentage of above 92%.

**Figure 7:**
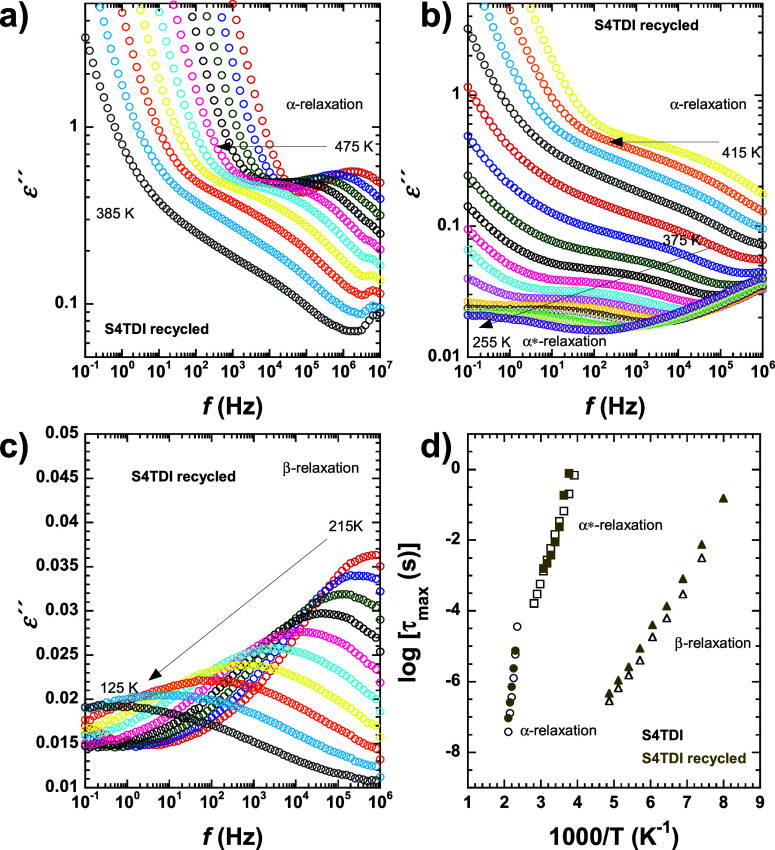
Isothermal plots of ε″ of the S4TDI recycled sample (a) at high temperatures, (b) at intermediate temperatures, and (c) at low temperatures. The characteristic times obtained from the maxima of the loss peaks are represented in panel d for the different relaxation processes observed (α-relaxation: filled circles; α*-relaxation: filled squares; β-relaxation: filled up-triangles) for the S4TDI original sample (black) and after the recycling process (olive green).

Once the confirmation that the structural properties remain unchanged was made, the dynamic behavior of both the original and the recycled materials was compared. For this purpose, BDS experiments were conducted on the recycled S4TDI sample, and the results are illustrated in Figure 7a,b,c.

As observed in this figure, mainly in the recycled sample, the same β-, α*-, and α-relaxations are still observed with remarkably similar dynamic behavior. To facilitate a direct comparison of the data between the original and recycled samples, a relaxation map is provided in Figure 7d. It confirms that both materials exhibit identical dynamic behavior, as the peaks of each relaxation process remain unchanged in the recycled material.

The strategy presented here introduces different avenues for addressing the formidable challenge of creating exceptional and optimal capacitors using innovative dielectric materials. These materials, rooted in dipolar glass vitrimers, present fresh opportunities owing to their elevated densities of polar groups possessing significant dipole moments capable of inducing pronounced orientational polarization in PTUs and thereby yielding high ε_r_′ values.

Due to the above, using these specimens as dielectric layers in capacitors would be well-supported, as it would allow the assembly of devices with an increased and more efficient energy storage process. More importantly, as a complement to the above, the vitrimer-like nature of these materials gives them the ability to self-repair, prolonging their own lifespan and, consequently, the lifespan of the as-prepared devices. In this sense, it is highly relevant to underscore the broad range of applications where capacitors find utility, from ensuring the correct operation of trivial circuits (e.g., household appliances, defibrillators, etc.) to more advanced and relevant ones such as power pulse applications, hybrid/electric vehicles, aerospace technology, robotics, (bio)sensors, rectifiers, and accumulators in wind and solar farms.

## 4 Conclusions

We have successfully prepared and characterized four different PTUs by combining various commercial monomers to study the influence of functionality (S3, S4) and the nature of substituents (HDI, TDI). Notably, the S4TDI system, reported for the first time in this work, is among them. These materials exhibit vitrimer-like behavior, characterized by short relaxation times and rapid complete relaxation when TDI is employed. The utilization of various complementary techniques has been crucial in elucidating the different transitions experienced by the materials when heated. The glass transition process is well-defined by calorimetry. Its complementarity with DMA and dielectric spectroscopy allows us to differentiate it from other relaxation transitions. Upon comparing the various prepared materials, we have noticed that the S4 systems exhibit higher *T*_g_ values in comparison to those prepared with S3, attributed to the greater number of cross-linking points observed in the S4 materials. Furthermore, systems containing TDI also demonstrate higher *T*_g_ values due to the hardness derived from this structure. In this context, among all the systems presented here, the S4TDI vitrimer-like material exhibits the highest *T*_g_ value. The slowing down of characteristic times in the S4TDI sample allows us to discern a different relaxation between the α- and β-relaxations. Another process is well-defined in the S4TDI sample, attributed to the unlocking of new dynamic motions within the system triggered by the activation of thiourethane bond exchange reactions. This process is in accordance with the *T*_v_ value observed by stress relaxation and creep analysis. The effects found here lead us to consider these materials as potential dipolar glass polymer systems, thanks to their reversibility, relatively high dielectric constants, and low loss factors. Coupled with the possibility of being recyclable materials, they become promising candidates for use as active layers in capacitors.

## Supporting Information Available

Composition and nomenclature of the formulations prepared; additional data obtained from TGA and DMA experiments; additional experimental BDS results for both isothermal and isochronal plots (PDF)
