# Supplementary material for: Revealing Dynamic Behavior in High Dielectric Poly(thiourethane)-Based Vitrimer-like Materials
Source: ACS Appl Polym Mater. 2024 Apr 27;6(9):5473–84. doi: 10.1021/acsapm.4c00681 (PMC11091852; doi:10.1021/acsapm.4c00681)
Supplement: Supplementary file 1 — ap4c00681_si_001.pdf [file ap4c00681_si_001.pdf]

Supporting Information

Revealing Dynamic Behavior in High Dielectric Poly(thiourethanes)-based vitrimer-like materials

Federico Guerrero-Ruiz[1], *Itziar Otaegi*[2], *Ester Verde-Sesto*[1,3], *Sebastian Bonardd*[1], *Jon Maiz*[1,3,*]

[1] Centro de Física de Materiales (CFM) (CSIC-UPV/EHU)-Materials Physics Center (MPC), Paseo Manuel de Lardizábal 5, 20018 Donostia-San Sebastián, Spain

[2] POLYMAT and Department of Advanced Polymers and Materials: Physics, Chemistry and Technology, Faculty of Chemistry, University of the Basque Country UPV/EHU, Paseo Manuel de Lardizábal 3, 20018, Donostia-San Sebastián, Spain

[3] IKERBASQUE-Basque Foundation for Science, Plaza Euskadi 5, 48009 Bilbao, Spain

Corresponding Author

* E-mail jon.maizs@ehu.eus

Table S1. Composition and nomenclature of the formulations prepared.

Material	Thiol (g)	Isocyanate (g)	DBTDL (mg)
**S3HDI**	1.53	0.97	73
**S4HDI**	1.48	1.05	76
**S3TDI**	1.51	0.99	72
**S4TDI**	1.46	1.04	75

Table S2. Main data obtained from thermogravimetric curves of prepared materials.

Material	T_i (K)[a]	Residue (%)[b]	T_{peak1} (K)	T_{peak2} (K)	T_{peak3} (K)
**S3HDI**	553	3.9	575	612	724
**S4HDI**	558	5.6	570	613	729
**S3TDI**	538	4.2	563	642	704
**S4TDI**	542	6.1	564	650	709

a. Onset degradation temperature.

b. Remaining weight percentage at 1023 K.

Table S3. T_v obtained from DMA analysis.

Material	DMA	
	**Stress relaxation (K)**	**Creep (K)**
**S3HDI**	387	384
**S4HDI**	354	366
**S3TDI**	406	385
**S4TDI**	371	381

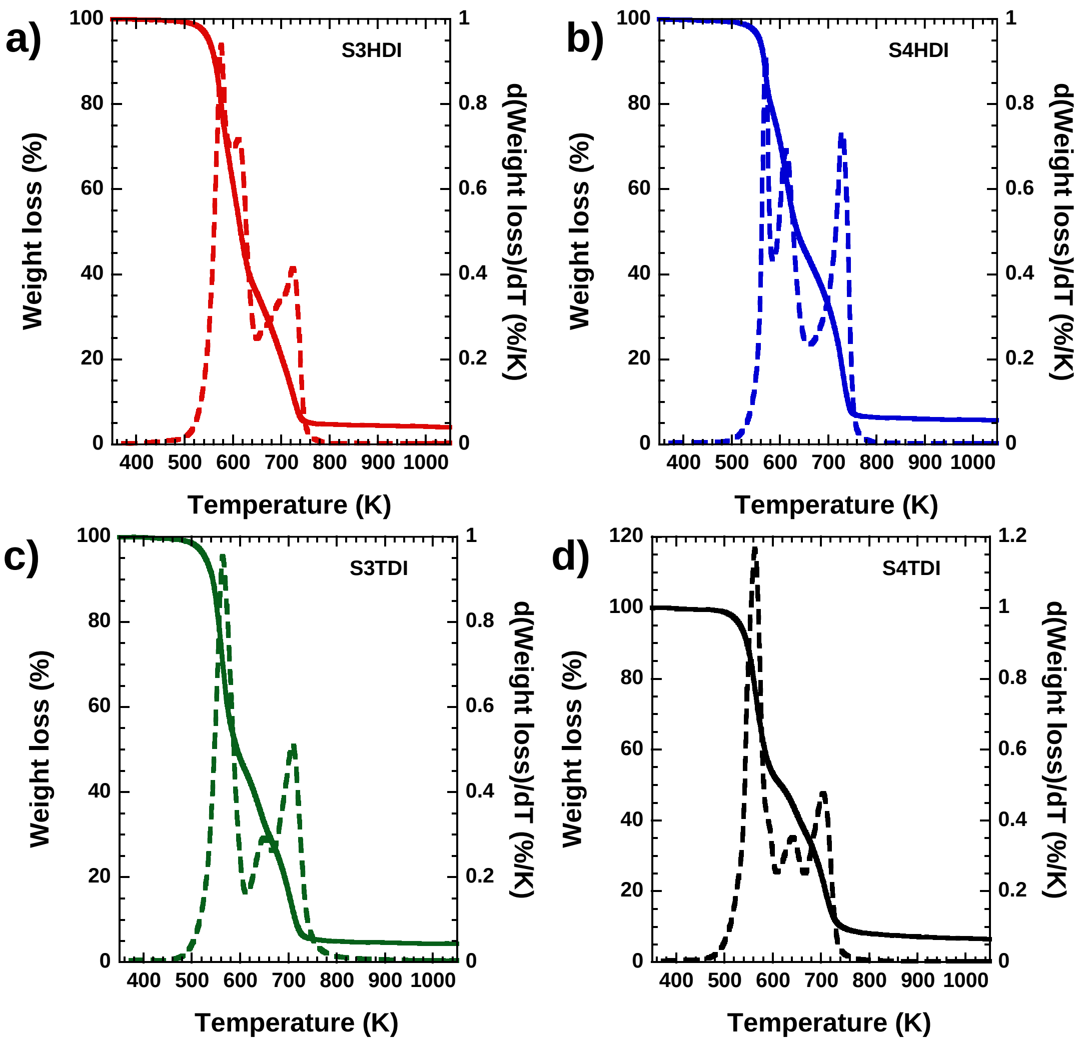

Figure S1. Thermogravimetric curves depicting weight loss and their derivative with respect to temperature for a) the S3HDI, b) the S4HDI, c) the S3TDI and d) the S4TDI samples.

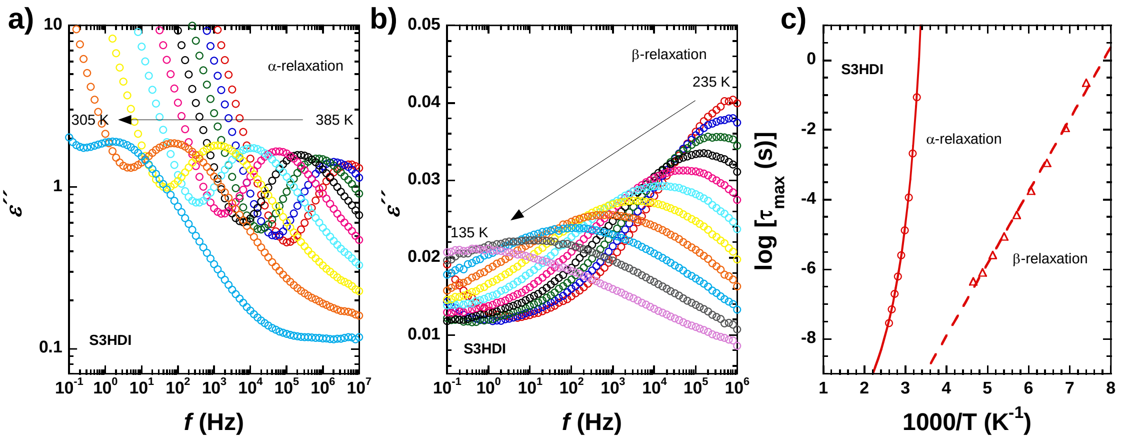

Figure S2. Isothermal plots of ε´´ S3HDI sample: a) at high temperatures, and b) at low temperatures. The characteristic times obtained from the maxima of the loss peaks are represented in c) for the different relaxation processes observed (α-relaxation: unfilled circles; β-relaxation: unfilled up-triangles).

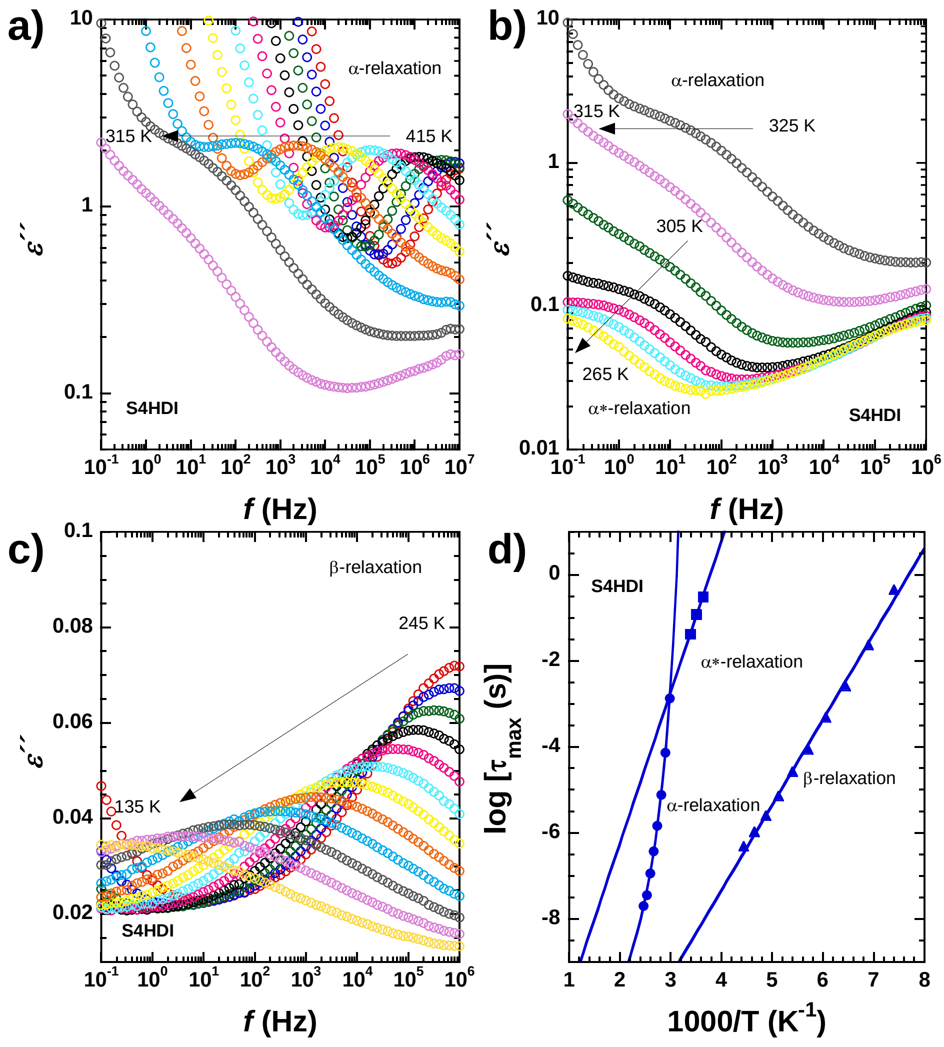

Figure S3. Isothermal plots of ε´´ S4HDI sample: a) at high temperatures, b) at intermediate temperatures, and c) at low temperatures. The characteristic times obtained from the maxima of the loss peaks are represented in d) for the different relaxation processes observed (α-relaxation: filled circles; α*-relaxation: filled squares; β-relaxation: filled up-triangles).

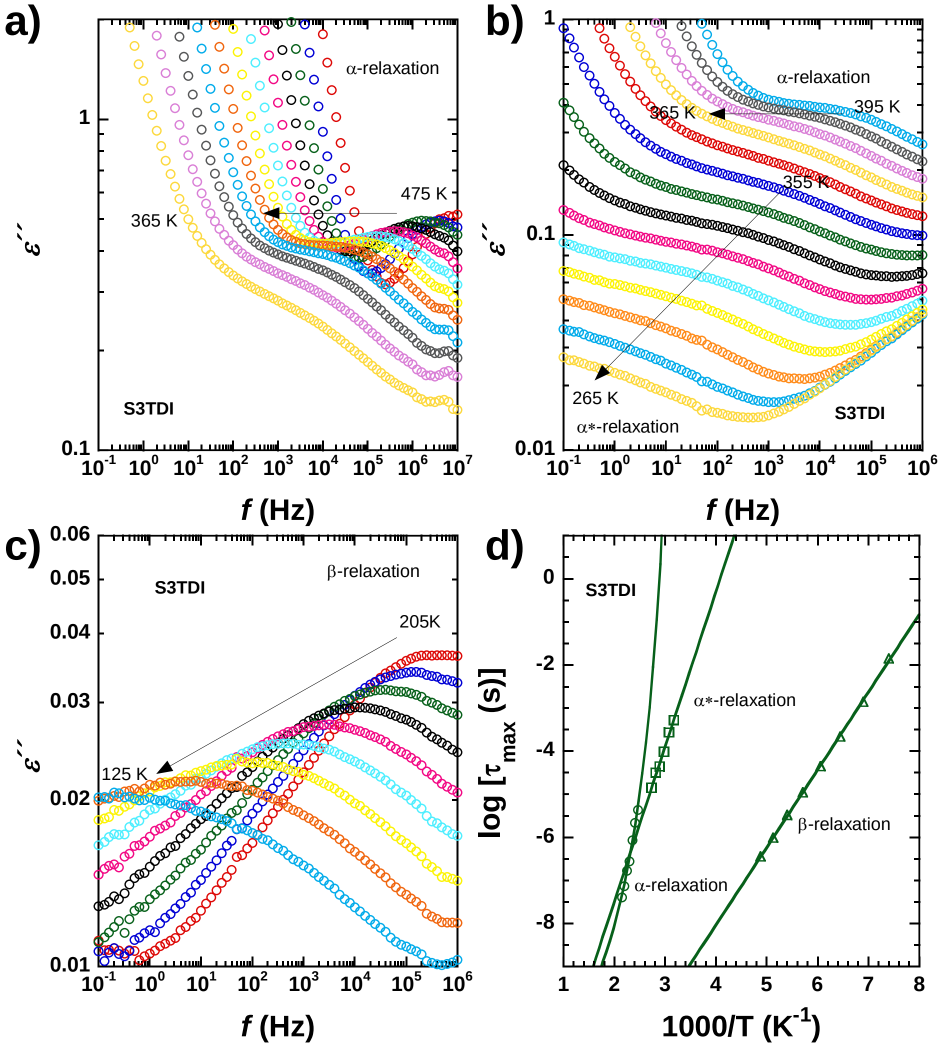

Figure S4. Isothermal plots of ε´´ S3TDI sample: a) at high temperatures, b) at intermediate temperatures, and c) at low temperatures. The characteristic times obtained from the maxima of the loss peaks are represented in d) for the different relaxation processes observed (α-relaxation: unfilled circles; α*-relaxation: unfilled squares; β-relaxation: unfilled up-triangles).

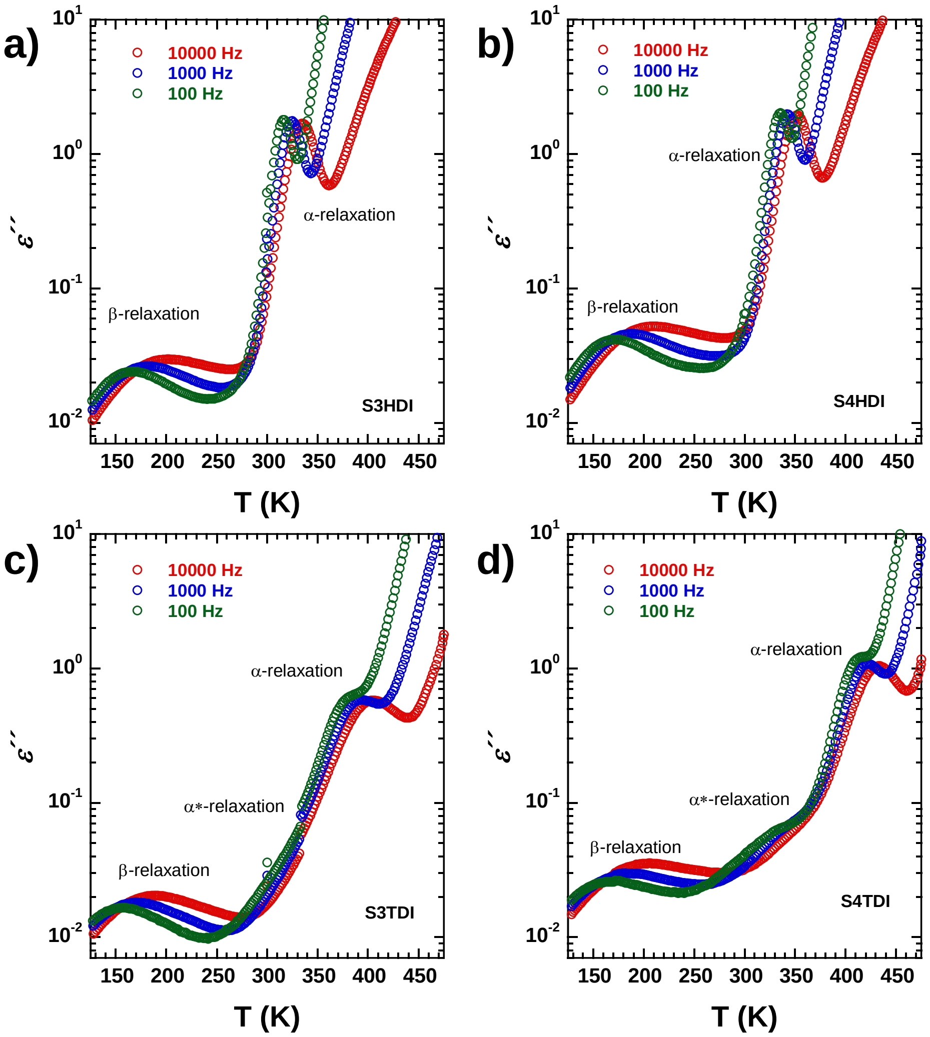

Figure S5. Isochronal plot of ε´´ a) S3HDI sample, b) S4HDI sample, c) S3TDI sample and d) S4TDI at three different frequencies: 10000 Hz (red), 1000 Hz (blue) and 100 Hz (green).

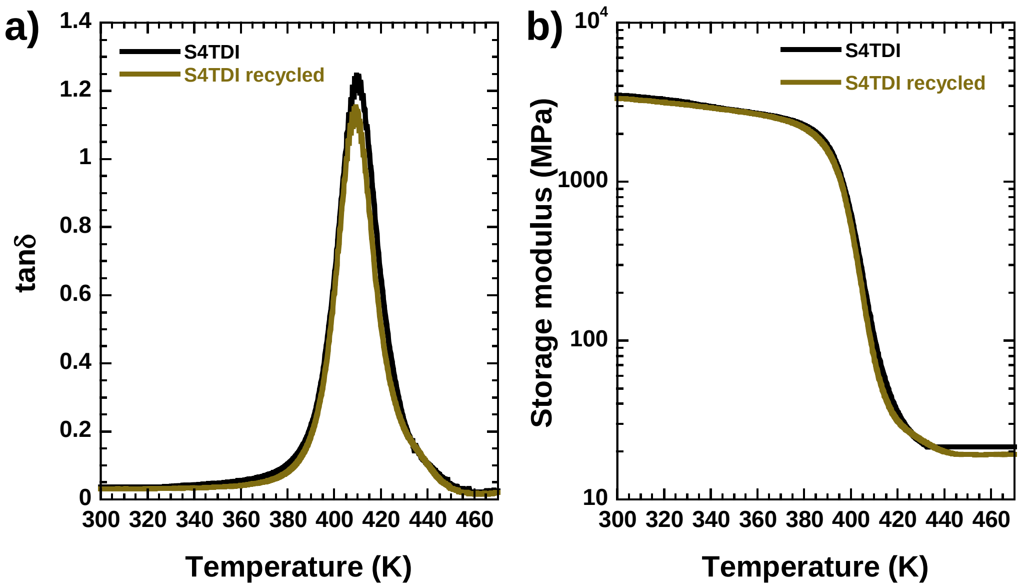

Figure S6. Plots of a) tanδ and b) storage moduli evolution with temperature of the original S4TDI sample (black) and after the recycling process (dark yellow).
